# Bam32/DAPP1-Dependent Neutrophil Reactive Oxygen Species in WKYMVm-Induced Microvascular Hyperpermeability

**DOI:** 10.3389/fimmu.2020.01028

**Published:** 2020-05-27

**Authors:** Li Hao, Aaron J. Marshall, Lixin Liu

**Affiliations:** ^1^Department of Anatomy, Physiology and Pharmacology, College of Medicine, University of Saskatchewan, Saskatoon, SK, Canada; ^2^Department of Immunology, University of Manitoba, Winnipeg, MB, Canada

**Keywords:** WKYMVm, microvascular permeability, neutrophils, reactive oxygen species, Bam32/DAPP1

## Abstract

B cell adaptor molecule of 32 kDa (Bam32), known as dual adapter for phosphotyrosine and 3-phosphoinositides 1 (DAPP1), has been implicated in regulating lymphocyte proliferation and recruitment during inflammation. However, its role in neutrophils during inflammation remains unknown. Using intravital microscopy, we examined the role of Bam32 in formyl peptide receptor agonist WKYMVm-induced permeability changes in post-capillary venules and assessed simultaneously neutrophil adhesion and emigration in cremaster muscles of Bam32-deficient (Bam32^−/−^) and wild-type (WT) control mice. We observed significantly reduced WKYMVm-induced microvascular hyperpermeability accompanied by markedly decreased neutrophil emigration in Bam32^−/−^ mice. The Bam32-specific decrease in WKYMVm-induced hyperpermeability was neutrophil-dependent as this was verified in bone marrow transplanted chimeric mice. We discovered that Bam32 was critically required for WKYMVm-induced intracellular and extracellular production of reactive oxygen species (ROS) in neutrophils. Pharmacological scavenging of ROS eliminated the differences in WKYMVm-induced hyperpermeability between Bam32^−/−^ and WT mice. Deficiency of Bam32 decreased WKYMVm-induced ERK1/2 but not p38 or JNK phosphorylation in neutrophils. Inhibition of ERK1/2 signaling cascade suppressed WKYMVm-induced ROS generation in WT neutrophils and microvascular hyperpermeability in WT mice. In conclusion, our study reveals that Bam32-dependent, ERK1/2-involving ROS generation in neutrophils is critical in WKYMVm-induced microvascular hyperpermeability during neutrophil recruitment.

## Introduction

Neutrophils, a group of polymorphonuclear leukocytes, are derived from hematopoietic stem cells, matured in and released from the bone marrow and circulate physiologically in the bloodstream (1, 2). In acute inflammation, one of the core events is the interaction between recruiting neutrophils and endothelial cells. This interaction elicits signaling changes in both cell types and regulates the endothelium barrier function through increasing adjacent endothelial spaces. Specifically, the neutrophil-endothelium interaction increases microvascular permeability through secreted neutrophil products, neutrophil adhesion and transmigration, and local respiratory bursts, such as the production of reactive oxygen species (ROS) (3).

B cell adaptor molecule of 32 kDa (Bam32), also known as dual adaptor for phosphotyrosine and 3-phosphoinositides 1 (DAPP1), is an intracellular protein widely expressed in almost all myeloid cells and lymphoid cells (4, 5). As an adaptor protein downstream of phosphoinositide 3-kinase (PI3K), Bam32 promotes B cell adhesion through regulating Rac1-mediated cytoskeletal rearrangement, actin remodeling, membrane ruffling, and lamellipodia formation (2, 3). It also integrates PI3K and Src kinase signals to promote germinal center progression in B cells and communication with T cells (6, 7). Beyond the PI3K signaling pathway, the role of Bam32 in the mitogen-activated protein kinase (MAPK) pathway was also reported in the activation of B cells (8). Bam32 is required in B cell proliferation and T cell-independent Ag responses of B cells (9, 10). In addition, Bam32 facilitates protein tyrosine kinase-mediated B cell Ag receptor internalization (11).

In other adaptive immune cell types, Bam32 is required for TCR-mediated cytokine production, proliferation, and actin-mediated spreading of CD4^+^ T cells (12). Bam32 in dendritic cells promotes the antigen-presenting functions and increases MHC class I-induced CD8^+^ T cell activation (13). Beyond its function in the adaptive immune cells, Bam32 constrains granule release through reducing FcεRI-induced calcium flux in mast cells (14). Although the roles of Bam32 have been established mostly in the adaptive immune cells and in mast cells during the past two decades, its role in many other innate immune cell types including neutrophils remains unclear.

Trp-Lys-Tyr-Met-Val-D-Met-NH_2_ (WKYMVm) is a synthetic agonist that mimics bacterial formyl peptide f-Met-Leu-Phe (fMLF) and activates leukocytes via formyl peptide receptors (FPR) (15, 16). WKYMVm preferentially stimulates FPR2 and is 1,000-fold more potent than natural bacterial peptide fMLF in triggering ROS production from mouse leukocytes (15, 17). WKYMVm also triggers the chemotactic migration of leukocytes as an end-target chemoattractant (18, 19). In addition to its effects on the immune system, WKYMVm functions in large vessels of cardiovascular system by reversing LPS-induced vascular hyporeactivity to phenylephrine in mouse aorta through decreasing the production of nitric oxide (20). One of our previous studies showed that PI3Kγ, an upstream regulator of Bam32 signaling in neutrophils, regulates WKYMVm-induced microvascular hyperpermeability (21). Other than that, the effect of WKYMVm on the crosstalk between the immune system and microvasculature is largely unknown.

In this study, we used intravital microscopy and fluorescence imaging *in vivo* to explore the role of Bam32 in WKYMVm-induced hyperpermeability in mouse post-capillary venules. We determined the effect of Bam32-dependent, ERK1/2-involving mechanism of ROS production in neutrophils on the change of microvascular barrier functions during neutrophil recruitment.

## Materials and Methods

### Animals

Bam32-deficient (Bam32^−/−^) mice were generated by Han et al. (4) on the C57BL/6 background and transferred to the University of Saskatchewan. Male mice between 6 and 12-week-old were used in the experiments along with age-matched male C57BL/6N mice (wild-type, WT) purchased from Charles River Canada (Saint-Constant, QC, Canada). This study was carried out with the protocol (#20070028) approved by the University Committee on Animal Care and Supply at the University of Saskatchewan and following the standards of the Canadian Council on Animal Care. All efforts were made to reduce animal suffering and all surgeries were performed under deep ketamine-xylazine anesthesia.

### Measurement of Microvascular Permeability, Neutrophil Adhesion, and Neutrophil Emigration

Jugular vein cannulation was performed on mice that were anesthetized after an intraperitoneal (i.p.) injection of a cocktail of ketamine (200 mg/kg, Roger, Montreal, QC, Canada) and xylazine (10 mg/kg, Bayer, Toronto, ON, Canada). The mouse cremaster muscle was surgically exposed as previously described (22, 23), and superfused with 37°C-warmed bicarbonate-buffered physiological saline (pH 7.4; containing in mM, NaCl 133.9, KCl 4.7, MgSO_4_ 1.2, and NaHCO_3_ 20.0; all reagents purchased from Fisher Scientific, Toronto, ON, Canada). The bright-field and fluorescence intravital microscopy was performed under an upright BX61WI Olympus microscope (Olympus, Tokyo, Japan) with an LUCPLFLN 20 × objective lens. FITC-labeled bovine serum albumin (BSA, 25 mg/kg, Sigma-Aldrich, Oakville, ON, Canada) was infused into the mouse circulation through the jugular vein 5 min prior to 1-h superfusion of exposed cremaster muscle with WKYMVm (0.1 μM, Phoenix Pharmaceutical, Burlingame, CA) (18) or the control saline. Fluorescence images were taken on the cremasteric venule every 5 min during superfusion with WKYMVm or the saline. The permeability index, calculated as the ratio of extravascular fluorescence intensity (FI) to the adjacent intravascular FI of the observed cremasteric venule, was measured as previously described (24, 25). The numbers of adherent and emigrated neutrophils were determined under bright-field microscopy prior to (0 min) and after 60-min superfusion with WKYMVm or the saline as described (23, 24). All the above reagents were prepared in 37°C-warmed bicarbonate-buffered physiological saline before superfusion. Where indicated, ERK1/2 inhibitor BVD-523 (Ulixertinib, 5 μM, Selleckchem, Houston, TX) or PD98059 (50 μM, Tocris, Minneapolis, MN) was pre-superfused on the cremaster muscle for 30 min prior to and throughout 60-min treatment with WKYMVm (26, 27).

### Harvest of Neutrophils From Mouse Bone Marrow

Murine neutrophils were freshly isolated from bone marrows of WT and Bam32^−/−^ mice. The femur and tibia were dissected immediately after each mouse was sacrificed, and the marrow cavity was flushed with ice-cold calcium- and magnesium-free phosphate-buffered saline (PBS). The bone marrow cells enriched in flushing fluid were separated by three-density (72, 64, and 52%) Percoll (GE Healthcare, Baie d'Urfe, QC, Canada) gradient centrifugation at 1,060 g for 30 min with a previously established method, yielding over 85% morphologically mature neutrophils (24).

### Bone Marrow Transplantation

To scrutinize the cell types where Bam32 played a role, we generated four different types of chimeric mice by transplantation of bone marrow from 5-week-old WT mice into 5-week-old WT and Bam32^−/−^ recipient mice (designated as WT → WT and WT → Bam32^−/−^, respectively) and of bone marrow from 5-week-old Bam32^−/−^ mice into 5-week-old WT and Bam32^−/−^ recipient mice (designated as Bam32^−/−^ → WT and Bam32^−/−^ → Bam32^−/−^, respectively). All recipients received two doses of X-ray irradiation (500 cGy/dose) with a 3-h interval, followed by injection of freshly prepared bone marrow cells (6–8 × 10^6^ cells) from either one of the two strains of donors through mouse tail veins. Thereafter, enrofloxacin (200 mg/L) freshly prepared in sterile drinking water along with sterile food was provided to the chimeric mice over the following 2 weeks. The mice were housed in sterile cages for 6 weeks after bone marrow transplantation to allow the reconstitution of full humoral immunity before experimental use.

### Determination of Intracellular ROS Generation

Freshly isolated mouse neutrophils from bone marrow were incubated in 37°C-warmed Krebs-Ringer phosphate glucose solution (containing in mM, NaCl 145, Na_2_HPO_4_ 5.7, KCl 4.86, CaCl_2_ 0.54, MgSO_4_ 1.22, and glucose 5.5) with 2,7-dichlorofluorescein diacetate (DCFDA, 20 μM, Sigma-Aldrich) with gentle agitation at 37°C for 30 min, with or without inhibitor diphenyleneiodonium (DPI, 40 μM, Sigma-Aldrich) (28, 29), BVD-523 (5 μM), or PD98059 (50 μM) where indicated. Neutrophils were washed thereafter and resuspended in 37°C DCFDA-free, inhibitor-containing Krebs-Ringer phosphate glucose solution. Fluorescence intensity was quantified with Fluoroskan Ascent Microplate Fluorometer (Thermo Fisher Scientific) upon and every 10 min after stimulation with WKYMVm (0.1 μM) or Phorbol 12-myristate 13-acetate (PMA, 0.2 μM, Sigma) (30).

### Determination of Extracellular Hydrogen Peroxide Generation

Bone marrow-derived mouse neutrophils were freshly isolated as described above and suspended in 37°C-warmed Krebs-Ringer phosphate glucose solution. An Amplex Red Hydrogen Peroxide assay kit (Thermo Fisher Scientific) was used to detect the production of extracellular hydrogen peroxide from neutrophils stimulated with WKYMVm (0.1 μM). All working solutions were prepared following the instructions provided by the manufacturer. Aliquots of neutrophils (1.0 × 10^6^/mL, 0.05 mL) were mixed with the working solution (volume ratio 1:1) in black 96-well plates upon and every 10 min after stimulation. Fluorescent signals were captured by Fluoroskan Ascent Microplate Fluorometer (Thermo Fisher Scientific).

### Rac1 Activation Assay

Fresh bone marrow-derived mouse neutrophils were prepared as described above and suspended in 37°C serum-free Hyclone RPMI 1640 (GE Healthcare). Colorimetric Rac1 G-LISA Activation Assay Kit (Cytoskeleton, Inc., Denver, CO) was used to quantitatively determine GTP-bound Rac1 after stimulation of neutrophils in suspension with WKYMVm (0.1 μM) at 37°C. All steps of lysing neutrophils and extracting GTP-bound Rac1 were performed strictly following the instruction provided by the manufacturer. Protein concentrations were determined using the Precision Red Advanced Protein Assay that came with the kit. Fluorescent signal of each sample on 96-well plates was captured by Fluoroskan Ascent Microplate Fluorometer (Thermo Fisher).

### Inhibition of ROS Generation *in vivo*

Mice were prepared for intravital microscopy and post-capillary venule permeability was determined in the cremaster muscle as described above. The NADPH oxidase inhibitor DPI (40 μM) and the hydrogen peroxide metabolizing enzyme catalase (200 units/mL, Sigma-Aldrich) (31) were prepared separately in 37°C-warmed bicarbonate-buffered saline. DMSO was used as the vehicle for DPI, whereas the inactive catalase prepared by boiling at 100°C for 10 min was used as the control for the catalase. The cremaster muscle was superfused with DPI or catalase for 30 min prior to and throughout the 60-min superfusion of WKYMVm. Fluorescence images were taken upon and every 5 min after WKYMVm was applied and permeability indices were determined at each time point as described above.

### Western Blotting

The levels of total p38, ERK1/2, SAPK/JNK MAPK, and their phosphorylated forms were determined using Western blotting. Briefly, freshly prepared bone marrow-derived mouse neutrophils were lysed in RIPA buffer, followed by mixing with 4 × Laemmli buffer (volume ratio 2:1) at 95°C for 5 min. The mixtures were resolved by 10% SDS-PAGE and transferred to a nitrocellulose membrane and immunoblotted as previously described (32). The nitrocellulose membrane was blocked with 5% BSA at room temperature for 1 h and then incubated at 4°C overnight with rabbit anti-phospho-p38, rabbit anti-phospho-ERK1/2, rabbit anti-phospho-SAPK/JNK (all three anti-phospho antibodies were used at 1:1,000 dilution; Cell Signaling Technology, Whitby, ON, Canada). After incubation with HRP-conjugated goat anti-rabbit secondary antibodies (1:4,000 dilution; Enzo, Burlington, ON, Canada), the blots were developed with Clarity ECL Substrates (Bio-rad, Montreal, QC, Canada). Thereafter, the developed blots were washed by stripping buffer (containing glycine 200 mM, sodium dodecyl sulfate 3.47 mM, and 1% Tween 20; pH 2.2) for 5 h and reprobed, respectively with rabbit anti-p38 (1:1,000 dilution; Cell Signaling Technology), rabbit anti-ERK1/2 (1:1,000 dilution; Cell Signaling Technology), rabbit anti-SAPK/JNK (1:1,000 dilution; Cell Signaling Technology) or mouse anti-β-actin (1:2,000 dilution; Invitrogen, Burlington, ON, Canada) antibodies, followed by incubation with corresponding secondary antibodies and Clarity ECL Substrates. The Image Studio Lite (Version 5.2, LI-COR Biotechnology, Lincoln, NE) was used for densitometric quantification of the detected bands.

### Statistical Analysis

Data are expressed as arithmetic means ± SEM from at least three independent experiments. Analyses of statistical differences among groups were performed using a two-tailed Student *t*-test or one-way ANOVA followed by the Holm-Sidak *post-hoc* analysis by using GraphPad Prism 7 (GraphPad Software, La Jolla, CA). *P* < 0.05 was considered to be statistically significant.

## Results

### Deficiency of Bam32 Decreases WKYMVm-Induced Microvascular Hyperpermeability and Neutrophil Emigration

**Figure 1:**
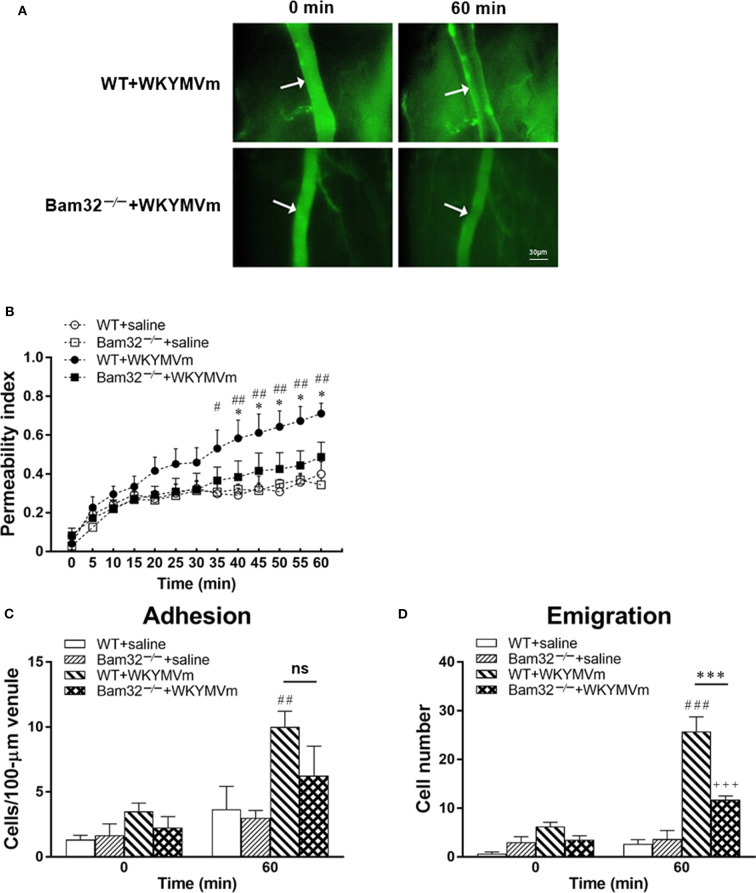
WKYMVm-induced microvascular hyperpermeability in WT and Bam32^−/−^ mice. **(A)** Representative fluorescence micrographs of hyperpermeability in mouse cremasteric post-capillary venules before (0 min) and 60 min after superfusion with WKYMVm in WT (upper panel) and Bam32^−/−^ (lower panel) mice. Arrow in micrograph indicates the venular segment for permeability index measurements (Magnification: 200×). **(B)** Permeability indices of mouse cremasteric post-capillary venules following 60-min superfusion with bicarbonate-buffered saline (white symbols) or WKYMVm (0.1 μM, black symbols) in WT and Bam32^−/−^ mice. Neutrophil adhesion number on 100-μm length of venule **(C)** and neutrophil emigration number (**D**, cells/443 × 286 μm^2^ field) were determined simultaneously in the same experiments of WKYMVm-induced cremasteric microvascular hyperpermeability before (0 min) and 60 min following superfusion with WKYMVm. **(B–D)** mean ± SEM, *n* = 4. Significant differences between WT and Bam32^−/−^ mice (*p < 0.05 and ***p < 0.001). Significant differences between WT mice with and without WKYMVm treatment (^#^*p* < 0.05, ^##^*p* < 0.01, and ^###^*p* < 0.001). Significant difference between Bam32^−/−^ mice with and without WKYMVm treatment (^+++^*p* < 0.001). ns, not significant.

To explore the role of Bam32 in post-capillary venule barrier functions and neutrophil recruitment, we utilized FPR agonist WKYMVm to induce microvascular leakage in WT and Bam32^−/−^ mice. Vascular permeability changes over time were assessed using intravital microscopy (Figure 1A). The baseline levels of microvascular leakage showed no significant difference between WT and Bam32^−/−^ mice during 60-min superfusion with the control saline (Figure 1B). After superfusion with WKYMVm, the microvascular leakage in WT mice was substantially increased, with significant elevations starting from 35 min as compared to the saline control. The leakage in Bam32^−/−^ mice were minimally increased but significantly less than that in WKYMVm-treated WT mice starting from 40 min. Because of the well-established function of WKYMVm in inducing neutrophil adhesion and emigration as an end-target chemoattractant (33), we simultaneously quantified the numbers of adherent and emigrated neutrophils before and after the 60-min WKYMVm treatment. Figures 1C,D showed that Bam32^−/−^ mice had a significantly lower number of emigrated neutrophils after 60 min treatment with WKYMVm, and a trend of reduced, albeit not significant, number of adherent neutrophils. These results reveal that Bam32 deficiency impairs neutrophil emigration and protects the integrity of microvascular barrier in mice in response to WKYMVm.

### Bam32 Deficiency in Hematopoietic Cells Is Sufficient to Impair WKYMVm-Induced Microvascular Hyperpermeability

**Figure 2:**
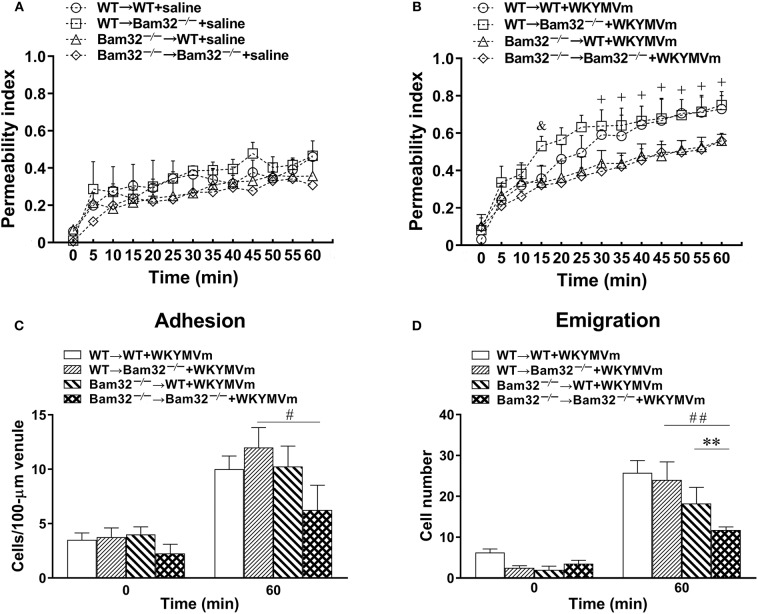
WKYMVm-induced microvascular hyperpermeability in bone marrow chimeric mice. Bone marrow chimeric mice were generated as described in *Material and Methods* for determination of the impact of Bam32 deficiency within hematopoietic (bone marrow derived) vs. radiation-resistant non-hematopoietic cells. Permeability indices of mouse cremasteric post-capillary venules following 60-min superfusion with bicarbonate-buffered saline **(A)** or WKYMVm (**B**, 0.1 μM) in chimeric mice. Neutrophil adhesion number on 100-μm length of venule **(C)** and neutrophil emigration number (**D**, cells/443 × 286 μm^2^ field) were determined simultaneously in the same experiments of WKYMVm-induced cremasteric microvascular hyperpermeability before (0 min) and 60 min following superfusion with WKYMVm. **(A–D)** mean ± SEM, *n* = 3. Significant difference between WT → WT and WT → Bam32^−/−^ mice (^&^*p* < 0.05). Significant differences between WT → WT and Bam32^−/−^ → WT mice (^+^*p* < 0.05). Significant difference between Bam32^−/−^ → WT and Bam32^−/−^ → Bam32^−/−^ mice (**p < 0.01). Significant differences between WT → Bam32^−/−^ and Bam32^−/−^ → Bam32^−/−^ mice (^#^*p* < 0.05 and ^##^*p* < 0.01).

Both neutrophils and endothelial cells contribute to chemoattractant-induced microvascular hyperpermeability. To distinguish whether the role of Bam32 in WKYMVm-induced microvascular leakage was in endothelial cells or bone marrow-derived recruiting neutrophils, we generated four types of bone marrow chimeric mice (WT → WT, WT → Bam32^−/−^, Bam32^−/−^ → WT, and Bam32^−/−^ → Bam32^−/−^). We assessed the microvascular leakage in these chimeric mice in response to WKYMVm and found no significant difference in microvascular permeability among four types of chimeric mice without stimulation with WKYMVm (Figure 2A). The microvascular permeability in WT → Bam32^−/−^ mice showed a trend of increase compared to WT → WT mice, which shared the same WT neutrophils but had different genotypes of endothelial background, and this reached significance at 15 min after superfusion with WKYMVm (Figure 2B). This difference appeared to diminish by 20 min, indicating Bam32 in endothelial cells might play a mild and transient role in elevating WKYMVm-induced microvascular leakage at early time points. Interestingly, after 30-min superfusion with WKYMVm, the microvascular permeability in WT → WT mice was significantly higher than that in Bam32^−/−^ → WT mice which had the same WT endothelial background without Bam32 in neutrophils. Similarly, the microvascular permeability in WT → Bam32^−/−^ mice was also significantly higher than that in Bam32^−/−^ → Bam32^−/−^ mice, demonstrating that Bam32 in neutrophils dominates in regulating WKYMVm-induced microvascular hyperpermeability regardless of the endothelium with or without Bam32. Figures 2C,D showed that the number of adherent neutrophils in Bam32^−/−^ → Bam32^−/−^ mice was significantly lower than that in WT → Bam32^−/−^ mice after 60-min superfusion with WKYMVm, revealing a possible role of neutrophil adhesion in regulating microvascular leakage. Moreover, the number of emigrated neutrophils in Bam32^−/−^ → Bam32^−/−^ mice was significantly lower than that in chimeric mouse strains with WT neutrophils (WT → WT, WT → Bam32^−/−^) after 60-min superfusion with WKYMVm, indicating that Bam32 in neutrophils is critically important in regulating WKYMVm-induced neutrophil emigration, whereas Bam32 in endothelial cells may play a secondary role in this process. These results from chimeric mice strongly suggest that Bam32 in neutrophils rather than in endothelial cells is required in WKYMVm-induced microvascular hyperpermeability.

### Bam32 Is Required for WKYMVm-Induced Intracellular and Extracellular ROS Production in Neutrophils

**Figure 3:**
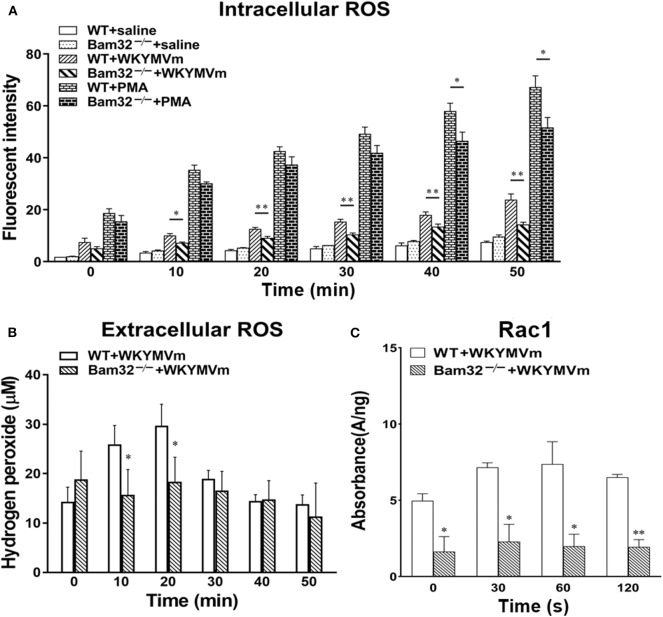
ROS generation and Rac1 activation in bone marrow-derived neutrophils from WT and Bam32^−/−^ mice. **(A)** Time course of intracellular generation of ROS in isolated neutrophils with saline, WKYMVm (0.1 μM) or PMA (0.2 μM). **(B)** Time course of extracellular generation of ROS in isolated neutrophils with WKYMVm. **(C)** Rac1 activation in isolated neutrophils with WKYMVm. **(A–C)** mean ± SEM, *n* = 3–4. Significant differences between WT and Bam32^−/−^ neutrophils (*p < 0.05 and **p < 0.01). Fluorescent signals at 0 min in **(A,B)** were measured immediately following the addition of saline, WKYMVm or PMA. Fluorescent signals at 0 min in **(C)** were measured in neutrophils without the treatment of WKYMVm.

Acute elevation in mouse microvascular permeability is attributed to many neutrophil-related factors, such as the production of ROS. A series of experiments were performed to determine the intracellular and extracellular production of ROS in bone marrow-derived neutrophils from WT and Bam32^−/−^ mouse strains *in vitro*. Figure 3A shows that deficiency of Bam32 significantly reduced the production of intracellular ROS in mouse neutrophils starting from 10-min stimulation with WKYMVm. Moreover, stimulation with PMA substantially increased the production of ROS in neutrophils but the differences between two mouse strains became significant only after 40-min stimulation with WKYMVm. This indicates that Bam32 may play a more important role in WKYMVm-induced rather than PMA-induced ROS generation in neutrophils. Moreover, extracellular ROS production in Bam32^−/−^ neutrophils was significantly lower than that in WT neutrophils at 10 min and 20 min following stimulation with WKYMVm (Figure 3B), further supporting the critical role of Bam32 in neutrophil ROS generation triggered by WKYMVm. As shown in Figure 3C, Bam32 deficiency also impaired the basal and WKYMVm-induced activation of Rac1. Together, these data suggest an unreported role for Bam32 in ROS generation in neutrophils.

### Bam32-Dependent Production of ROS Promotes WKYMVm-Induced Microvascular Hyperpermeability

**Figure 4:**
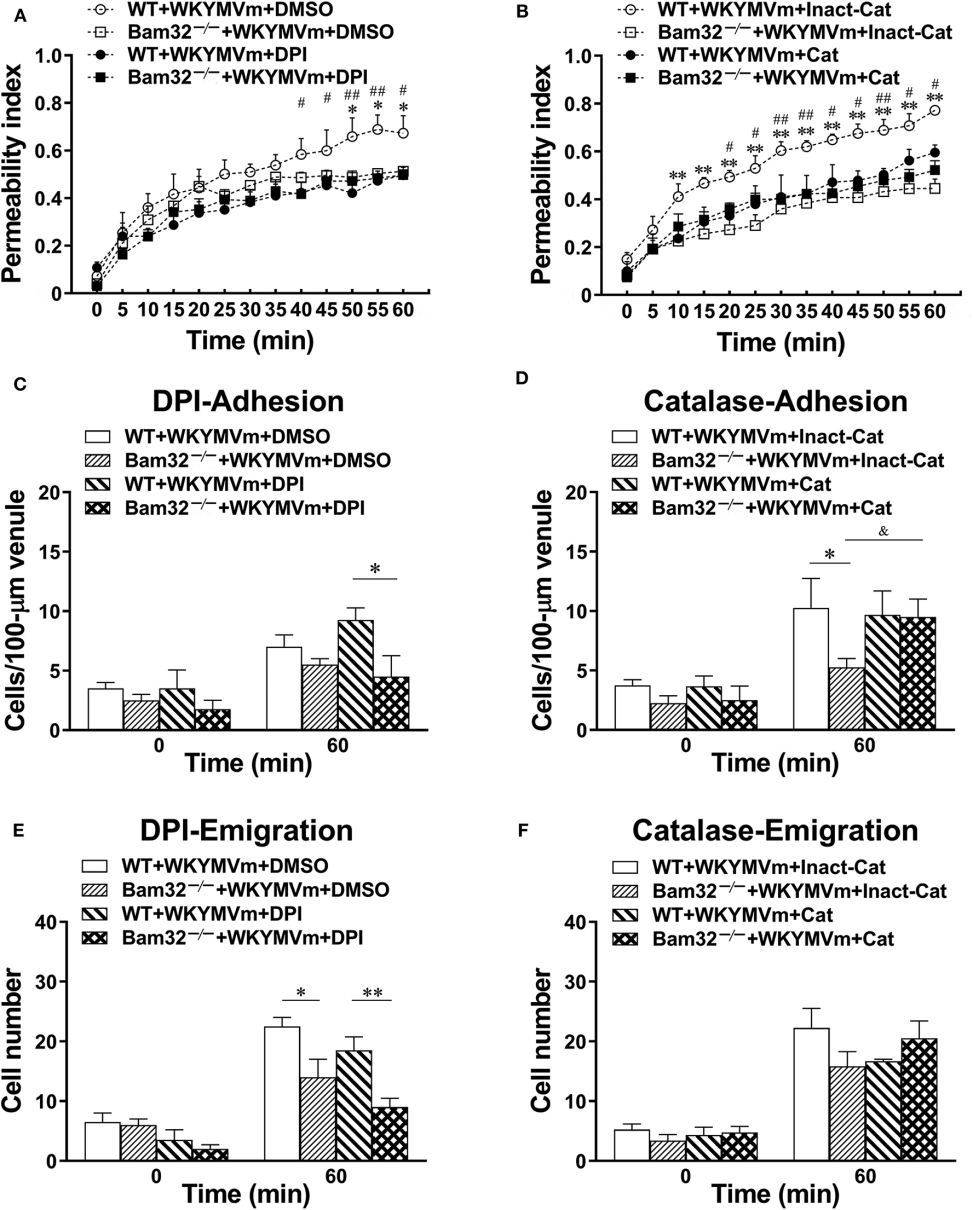
Pharmacological inhibition of reactive oxygen species generation in WKYMVm-induced microvascular hyperpermeability. **(A)** Permeability indices of mouse cremasteric post-capillary venules following 60-min WKYMVm (0.1 μM) superfusion in the presence of vehicle (DMSO) or DPI (40 μM) for 30 min prior to and during 60-min WKYMVm superfusion. **(B)** Permeability indices of mouse cremasteric post-capillary venules following 60-min WKYMVm superfusion in the presence of inactivated catalase (Inact-Cat) or functional catalase (Cat, 200 units/mL) for 30 min prior to and during 60-min WKYMVm superfusion. Neutrophil adhesion number of 100-μm length of venule **(C)** and neutrophil emigration number (**E**, cells/443 × 286 μm^2^ field) were determined simultaneously in the same experiments of WKYMVm-induced cremasteric microvascular hyperpermeability before (0 min) and 60 min following superfusion with WKYMVm in the presence of DMSO or DPI. Neutrophil adhesion number of 100-μm venule **(D)** and neutrophil emigration number (**F**, cells/443 × 286 μm^2^ field) were determined simultaneously in the same experiments of WKYMVm-induced cremasteric microvascular hyperpermeability before (0 min) and 60 min following superfusion with WKYMVm in the presence of inactivated catalase or functional catalase. **(A–F)** mean ± SEM, *n* = 4–6. Significant differences between WT and Bam32^−/−^ mice (*p < 0.05 and **p < 0.01). Significant differences between WT mice with or without DPI or functional catalase (^#^*p* < 0.05 and ^##^*p* < 0.01). Significant difference between Bam32^−/−^ mice with functional catalase and with inactivated catalase (^&^*p* < 0.05).

To verify the importance of Bam32-dependent ROS production in WKYMVm-induced mouse microvascular leakage, we applied an NADPH oxidase inhibitor DPI or a hydrogen peroxide metabolizing enzyme catalase to WKYMVm-superfused mouse cremaster muscle. As depicted in Figure 4A, WKYMVm-induced microvascular hyperpermeability in WT mice was again found to be significantly higher than that in Bam32^−/−^ mice; however, the application of DPI alleviated the microvascular leakage in WT mice and completely eliminated the differences in permeability changes between these mouse strains. Similarly, the application of catalase not only substantially reduced the microvascular leakage in WT mice, but also eliminated the differences in permeability changes between two mouse strains (Figure 4B). In contrast, DPI treatment did not impact the numbers of adherent and emigrated neutrophils in WT or Bam32^−/−^ mice, whereas deficiency of Bam32 significantly decreased the numbers of adherent and emigrated neutrophils after 60-min of WKYMVm treatment when DPI was present (Figures 4C,E). Moreover, the application of catalase did not significantly change the numbers of adherent and emigrated neutrophils induced by WKYMVm in either mouse strain, except for increased number of adherent neutrophils in WKYMVm-treated Bam32^−/−^ mice for unknown reason (Figures 4D,F). These results reveal that Bam32-dependent ROS production in recruiting neutrophils, functionally independent of neutrophil adhesion, and emigration, is critically required in WKYMVm-induced microvascular hyperpermeability.

### Deficiency of Bam32 Impairs WKYMVm-Induced Phosphorylation of ERK1/2 in Neutrophils Without Influencing Phosphorylation of p38 and JNK

**Figure 5:**
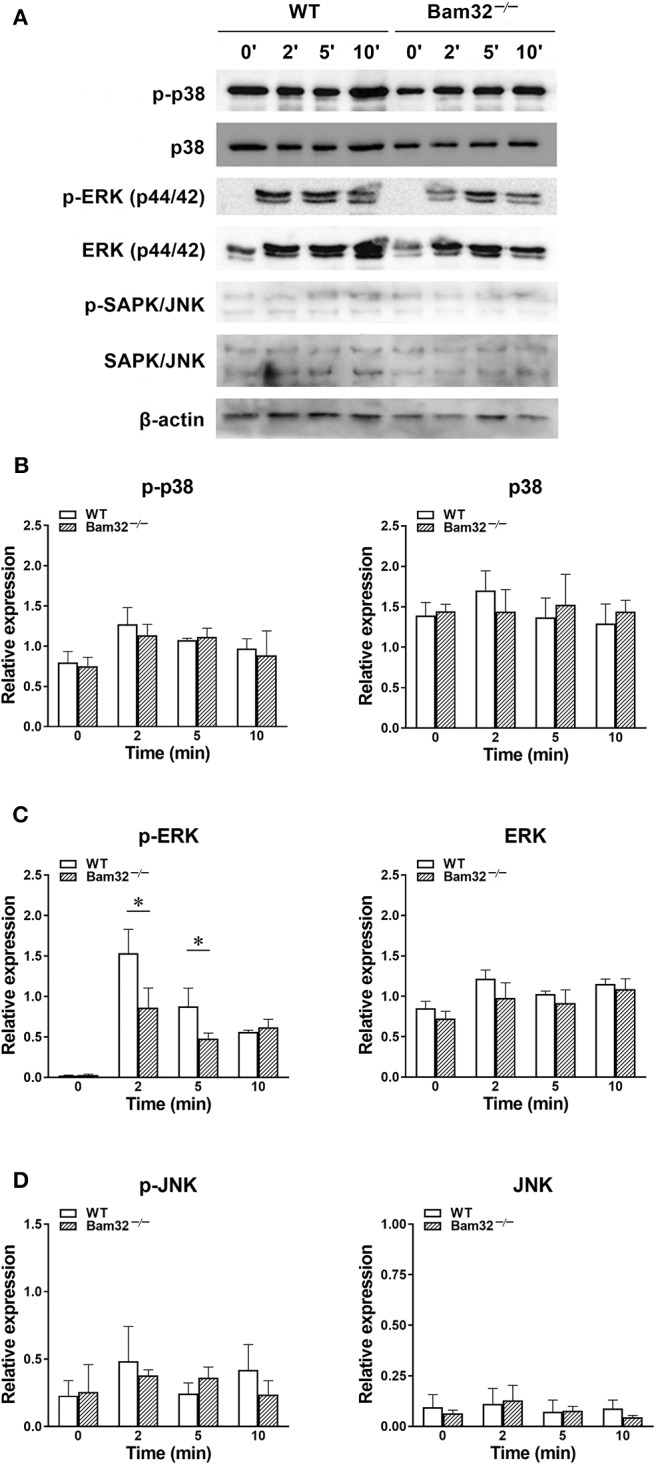
WKYMVm-induced phosphorylation of p38, ERK1/2 and JNK MAPK in isolated WT and Bam32^−/−^ neutrophils. **(A)** Representative original Western blots of phosphorylated and total p38, ERK1/2, and JNK MAPK determined in bone marrow-derived neutrophils from WT and Bam32^−/−^ mice before (0 min) and 2–10 min after treatment with WKYMVm (0.1 μM). Levels of phosphorylated (p) and total p38 **(B)**, ERK1/2 **(C)**, and JNK **(D)** determined in bone marrow-derived neutrophils from WT and Bam32^−/−^ mice before (0 min) and 2–10 min after treatment with WKYMVm (0.1 μM). Mean ± SEM, *n* = 3. *Significant differences between WT and Bam32^−/−^ neutrophils.

Given that WKYMVm activates three downstream MAPK cascades, namely p38, ERK1/2, and JNK through FPR (34), we further tested the possible involvement of these MAPK signaling pathways in Bam32^−/−^ neutrophils. As shown in Figures 5A–D, deficiency of Bam32 failed to impact the phosphorylation or total levels of p38 or JNK, but significantly impaired the phosphorylation of ERK1/2 after 2-min and 5-min stimulation with WKYMVm. These results indicate that the impairment of ERK1/2 signaling pathway may be involved in Bam32-dependent production of ROS in WKYMVm-stimulated mouse neutrophils.

### Pharmacological Inhibition of the ERK1/2 Signaling Pathway Suppresses WKYMVm-Induced Intracellular ROS Production *in vitro* and Microvascular Hyperpermeability *in vivo*

**Figure 6:**
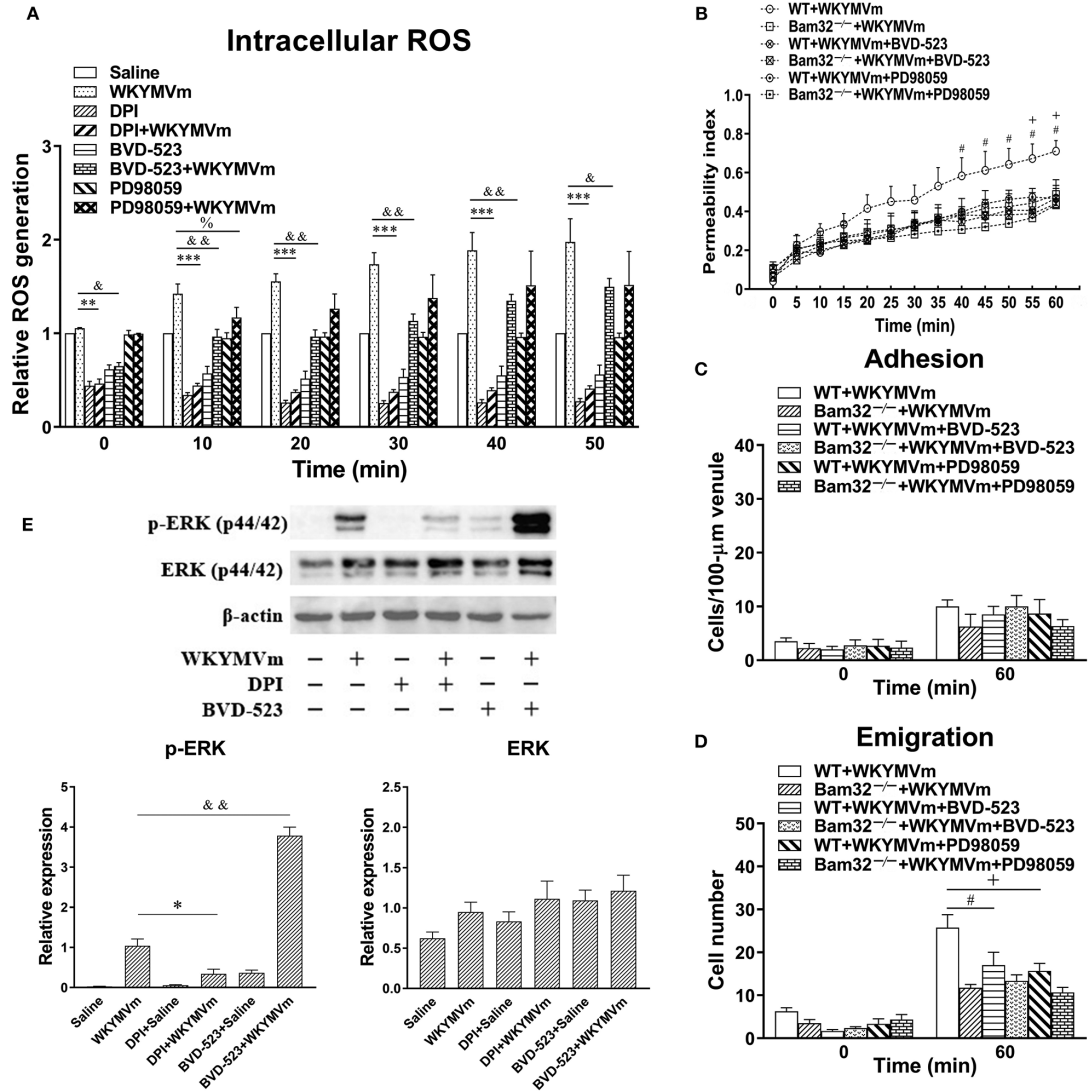
Pharmacological inhibition of ERK1/2 signaling in WKYMVm-induced intracellular ROS generation and microvascular hyperpermeability. **(A)** Time course of intracellular generation of ROS in isolated neutrophils pre-incubated with vehicle (DMSO), DPI (40 μM), BVD-523 (5 μM), and PD98059 (50 μM), respectively, for 30 min prior to and during 60-min treatment of WT neutrophils with saline or WKYMVm (0.1 μM). **(B)** Permeability indices of mouse cremasteric post-capillary venules following 60-min WKYMVm superfusion in the absence or presence of BVD-523 or PD98059 for 30 min prior to and during 60-min WKYMVm superfusion in WT and Bam32^−/−^ mice. Neutrophil adhesion number on 100-μm length of venule **(C)** and neutrophil emigration number (cells/443 × 286 μm^2^ field, **D**) were determined simultaneously in the same experiments of WKYMVm-induced cremasteric microvascular hyperpermeability before (0 min) and 60 min following superfusion with WKYMVm in the absence or presence of BVD-523 or PD98059 in the two mouse strains. **(E)** Original Western blots representative of at least three independent experiments and the quantification data of phosphorylated and total ERK1/2 determined in bone marrow-derived WT neutrophils pre-incubated with vehicle, DPI or BVD-523 for 30 min prior to the 2-min treatment of WT neutrophils with saline or WKYMVm. **(A–E)** mean ± SEM, *n* = 3–4. Significant differences between WKYMVm-treated WT neutrophils with and without DPI (*p < 0.05, **p < 0.01, and ***p < 0.001). Significant differences between WKYMVm-treated WT neutrophils with and without BVD-523 (^&^*p* < 0.05 and ^&&^*p* < 0.01). Significant difference between WKYMVm-treated WT neutrophils with and without PD98059 (^%^*p* < 0.05). Significant differences between WKYMVm-treated WT mice with and without BVD-523 (^#^*p* < 0.05). Significant differences between WKYMVm-treated WT mice with and without PD98059 (^+^*p* < 0.05).

To verify the possible involvement of ERK1/2 signaling in neutrophil ROS generation and microvascular leakage, we determined the effects of the NADPH oxidase inhibitor DPI, the ERK1/2 signaling inhibitors BVD-523 and PD98059 on WKYMVm-induced intracellular ROS generation, microvascular hyperpermeability, and ERK1/2 phosphorylation. We found that DPI and BVD-523 significantly reduced basal and WKYMVm-induced generation of intracellular ROS in WT neutrophils at all time points tested, whereas PD98059 only suppressed WKYMVm-triggered ROS generation at 10 min (Figure 6A), indicating the involvement of ERK1/2 activation in neutrophil ROS generation at early time. Moreover, pre-superfusion of mouse cremaster muscle with BVD-523 or PD98059 alleviated WKYMVm-induced microvascular hyperpermeability starting from 40 and 55 min, respectively (Figure 6B), and reduced the number of emigrated neutrophils in WKYMVm-treated WT mice without changing the number of adherent neutrophils (Figures 6C,D), supporting that the ERK1/2-involving ROS generation regulates WKYMVm-induced microvascular hyperpermeability. Interestingly, we found that inhibition of ROS production by DPI resulted in debilitated ERK1/2 phosphorylation in WKYMVm-stimulated WT neutrophils (Figure 6E), suggesting that ROS production may also contribute to activation of the ERK1/2 signaling pathway. The application of BVD-523 markedly increased WKYMVm-induced ERK1/2 phosphorylation (Figure 6E), consistent with previously published data showing that this inhibitor increases ERK1/2 phosphorylation while strongly inhibiting its activity in phosphorylating substrates (26). In brief, these results strongly suggest that Bam32-dependent activation of ERK1/2 signaling pathway is crucial in WKYMVm-induced neutrophil ROS generation and subsequent neutrophil ROS-mediated increases in microvascular leakage.

## Discussion

In this study, we unravel a novel mechanism for Bam32-dependent production of ROS through ERK1/2 signaling by neutrophils with functional impact in WKYMVm-induced microvascular hyperpermeability. Since the endothelium *per se* has the capability of inducing local oxidative stress to impair the microvascular barrier, we scrutinized the role of Bam32 in neutrophils by determining the microvascular hyperpermeability using bone marrow chimeric mice. Moreover, we found the evidence that Bam32 plays a role in WKYMVm-induced activation of ERK1/2 signaling via formyl peptide receptors, leading to ROS production and microvascular leakage. Therefore, our study reveals that Bam32-mediated changes in WKYMVm-induced microvascular hyperpermeability are largely due to Bam32-dependent, ERK1/2-involving production of ROS by the recruiting neutrophils.

The proinflammatory effects of WKYMVm have been reported previously (35–37). We show in this study that this formyl peptide receptor agonist also elevates microvascular leakage in WT mice. Deficiency of Bam32 significantly lowered hyperpermeability in WKYMVm-treated mouse microvasculature especially at late time points (after 40 min), but increased microvascular hyperpermeability at 15–25 min in chemokine CXCL2-treated mouse microvasculature (data shown in Figure S1). The role of Bam32 in WKYMVm-induced vascular hyperpermeability clearly impacts the recruiting neutrophils because a significantly lower number of emigrated neutrophils were found simultaneously in the same segment of venule where the microvascular permeability was determined. The neutrophil-intrinsic function of Bam32 is supported by the results from bone marrow chimeric mice with WKYMVm-superfused cremasteric vasculature. Chimeric mice with Bam32-deficient neutrophils, regardless of their endothelial background, had decreased microvascular hyperpermeability, strongly indicating that the role of Bam32 in neutrophils is sufficient to significantly impact WKYMVm-induced venule barrier functions in response to WKYMVm. Interestingly, a higher level of leakage in WT → Bam32^−/−^ mice than in WT → WT mice was noticeable at 15-min time point after superfusion with WKYMVm. Although this difference was transient and only appears in chimeric mice with WT neutrophils, it suggests the possibility that a subordinate Bam32-mediated mechanism for regulating microvascular leakage may exist in endothelial cells.

Neutrophil-dependent local production of pro-inflammatory mediators, such as oxidants (38), impairs vascular barrier function through various mechanisms. Previous studies reported that ROS increased the phosphorylation of catenin in endothelial cells, facilitating the internalization of VE-cadherin and opening of adherens junctions (39). Moreover, ROS regulate actin rearrangement in endothelial cells through Rac1 activation to increase microvascular permeability (40). In addition, ROS damage tight junctions of endothelium and increase the vascular leakage by inducing redistribution of the transmembrane proteins (41). In our study, the production of intracellular and extracellular ROS in WKYMVm-stimulated neutrophils was impaired by the deficiency of Bam32. Our finding that NADPH oxidase inhibitor DPI and hydrogen peroxide metabolizing enzyme catalase blocked WKYMVm-induced increases in microvascular permeability suggests a critical functional role of Bam32-dependent ROS production in WKYMVm-induced increases in mouse microvascular barrier functions. In contrast, chemokine CXCL2 failed to induce differences in ROS generation between WT neutrophils and Bam32^−/−^ neutrophils even after TNFα priming (data shown in Figure S2), indicating that deficiency of Bam32 increases CXCL2-induced microvascular hyperpermeability through a completely ROS-independent mechanism.

As prerequisite events to the local ROS production of recruiting neutrophils, neutrophil adhesion, and emigration trigger microvascular leakage through physical interactions with the endothelium at the circulation and tissue interface (42, 43). However, our data show that the role of Bam32 in mediating microvascular leakage may be due to Bam32-dependent production of ROS in neutrophils, rather than Bam32-dependent emigration. This is because, after inhibition of ROS production by DPI in WKYMVm-treated mice, the persisting significant gap in the numbers of adherent and emigrated neutrophils was insufficient to induce Bam32-dependent differences in microvascular hyperpermeability. This uncoupling of neutrophil adhesion and emigration to microvascular leakage was also reported in previous studies. Leukotriene B_4_-induced transmigrating neutrophils infiltrated into alveolar spaces of human lungs without increasing microvascular permeability (44). The formation of the endothelial dome structure, also known as the transmigratory cup, encapsulated the transmigrating neutrophils, and minimized the microvascular leakage (45, 46). Therefore, our study suggests that Bam32-dependent increases in WKYMVm-induced microvascular permeability are attributed to the alteration in Bam32-dependent production of ROS in recruiting neutrophils.

Rac1 is mainly referred in regulating the polarization and directionality in neutrophil chemotaxis (47, 48), but also involved in NADPH oxidase-mediated ROS generation in neutrophils (49). Given the possible involvement of Rac1 in WKYMVm-induced neutrophil ROS production, we determined the Rac1 activation in WKYMVm-stimulated neutrophils. Interestingly, our results show that Bam32 was required in WKYMVm-induced Rac1 activation. Although our study suggests a role for Bam32 in NADPH oxidase-mediated ROS generation, this Bam32-dependent functional connection between Rac1 activation and ROS generation may require further verification.

Bam32, albeit well-established as an adaptor molecule downstream of PI3K signaling pathway, also plays a role in mediating MAPK signals in immune cells. In B cells Bam32 was found to regulate phosphorylation of ERK and JNK (4, 10). Moreover, Bam32 in T cells is important for optimal TCR-mediated ERK1/2 activation (12). In this study, we revealed a novel role of Bam32 in modulating ERK1/2 signaling without influencing p38 and JNK in WKYMVm-stimulated neutrophils. We further demonstrated that signaling via ERK1/2 is required for WKYMVm-induced ROS production and subsequent microvascular leakage based on our experiments using BVD-523, an ATP-competitive and reversible covalent inhibitor of ERK1/2 kinases, and PD98059, a highly selective inhibitor of MEK1 activation in the MAPK signaling cascade. The application of these two ERK1/2 inhibitors showed similar trend in impairing neutrophil ROS generation and microvascular hyperpermeability in response to WKYMVm and supported the role of ERK1/2 signaling in Bam32-dependent ROS generation and microvascular permeability changes. It is worth noting that the data of BVD-523 inhibition should be taken cautiously because of the possibly non-specific inhibition of PMA-induced, protein kinase C-involving neutrophil ROS production unraveled in this study (data shown in Figure S3).

A previous study found that the activation of ERK1/2 signaling results in conformational changes in p47^phox^, an important component of NADPH oxidase for ROS production (50), indicating a potential mechanism linking ERK with ROS production. Reciprocally, the production of ROS modulates ERK1/2 phosphorylation (51, 52), suggesting that the low production of ROS in Bam32-deficient neutrophils may, in turn, contribute to reduced ERK1/2 phosphorylation in WKYMVm-stimulated neutrophils. Given this bidirectional cross-talk between ERK activation and ROS production, further explorations will be needed to reveal the interactions between Bam32-dependent ROS production and Bam32-dependent ERK1/2 signaling in the process of microvascular leakage.

## Conclusion

Bam32 is required for WKYMVm-induced hyperpermeability in mouse cremasteric post-capillary venule through enhancing the generation of extracellular and intracellular ROS from neutrophils. This role of Bam32 in neutrophils for microvascular hyperpermeability is not functionally linked to neutrophil adhesion and emigration. Bam32-mediated ERK1/2 signaling is involved in the production of ROS induced by WKYMVm and the inhibition of either ERK1/2 or ROS can block WKYMVm-induced hyperpermeability. Our study provides a mechanistic insight into the role of Bam32 in neutrophils for regulating microvascular leakage in acute inflammation where formyl peptide receptors are involved.

## Data Availability Statement

All datasets generated for this study are included in the article/Supplementary Material.

## Ethics Statement

This animal study was reviewed and approved by University Committee on Animal Care and Supply at the University of Saskatchewan.

## Author Contributions

LH conducted experiments. LH and LL designed research and performed data analysis and interpretation. LH, AM, and LL wrote and revised manuscript. All authors read and approved the final manuscript.

## Conflict of Interest

The authors declare that the research was conducted in the absence of any commercial or financial relationships that could be construed as a potential conflict of interest.

## Supplementary Material

Figure S1 CXCL2-induced microvascular hyperpermeability in WT and Bam32^−/−^ mice. **(A)** Permeability indices of mouse cremasteric post-capillary venules following 60-min superfusion with bicarbonate-buffered saline (white symbols) or CXCL2 (0.5 nM, black symbols) in the two mouse strains. Neutrophil adhesion number on 100-μm length of venule **(B)** and neutrophil emigration number (**C**, cells/443 × 286 μm^2^ field) determined simultaneously in the same experiments of CXCL2-induced cremasteric microvascular hyperpermeability prior to (0 min) and 60 min following superfusion with CXCL2. **(A–C)**, mean ± SEM, *n* = 4. Significant differences between WT and Bam32^−/−^ mice (^*^*p* < 0.05 and ^**^*p* < 0.01). Significant differences between WT mice with and without CXCL2 treatment (^#^*p* < 0.05 and ^##^*p* < 0.01).

Figure S2 Time course of CXCL2 (0.5 nM)-induced generation of intracellular ROS in isolated neutrophils primed with or without TNFα (12 pM). Mean ± SEM, *n* = 4.

Figure S3 Effects of ERK1/2 inhibitor BVD-523 on intracellular ROS production in WT neutrophils treated with WKYMVm (0.1 μM) or PMA (0.2 μM). Mean ± SEM, *n* = 3–4. Significant differences between WKYMVm-stimulated groups with and without BVD-523 (^*^*p* < 0.05, ^**^*p* < 0.01 and ^***^*p* < 0.001). Significant differences between PMA-stimulated groups with and without BVD-523 (^###^*p* < 0.001).
