# Supplementary figures and images for: Bam32/DAPP1-Dependent Neutrophil Reactive Oxygen Species in WKYMVm-Induced Microvascular Hyperpermeability
Source: Front Immunol. 2020 May 27;11:1028. doi: 10.3389/fimmu.2020.01028 (PMC7267069; doi:10.3389/fimmu.2020.01028)

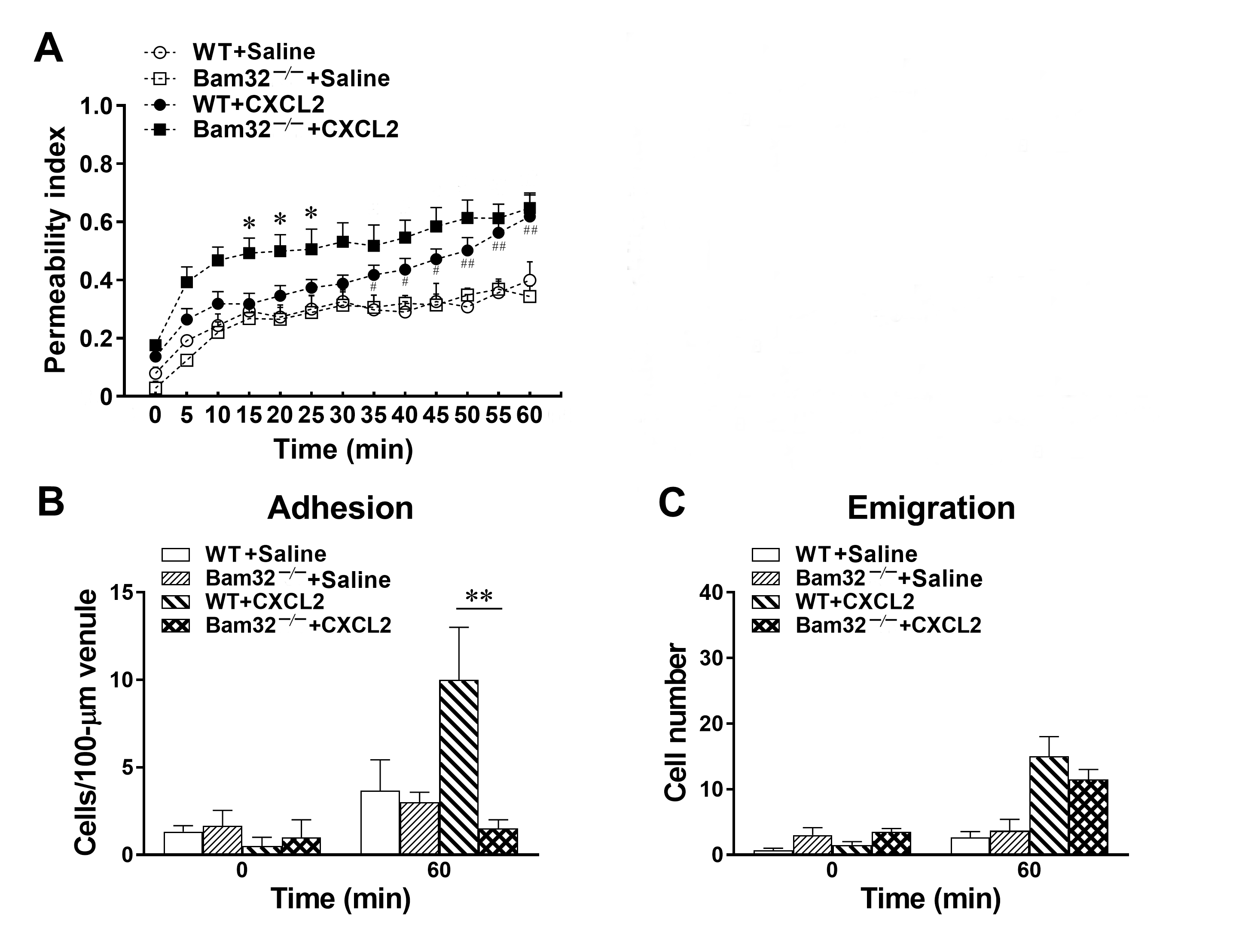

Supplement: Figure S1 — CXCL2-induced microvascular hyperpermeability in WT and Bam32−/− mice. (A) Permeability indices of mouse cremasteric post-capillary venules following 60-min superfusion with bicarbonate-buffered saline (white symbols) or CXCL2 (0.5 nM, black symbols) in the two mouse strains. Neutrophil adhesion number on 100-μm length of venule (B) and neutrophil emigration number (C, cells/443 × 286 μm2 field) determined simultaneously in the same experiments of CXCL2-induced cremasteric microvascular hyperpermeability prior to (0 min) and 60 min following superfusion with CXCL2. (A–C), mean ± SEM, n = 4. Significant differences between WT and Bam32−/− mice (*p < 0.05 and **p < 0.01). Significant differences between WT mice with and without CXCL2 treatment (#p < 0.05 and ##p < 0.01). [file Image_1.TIF]

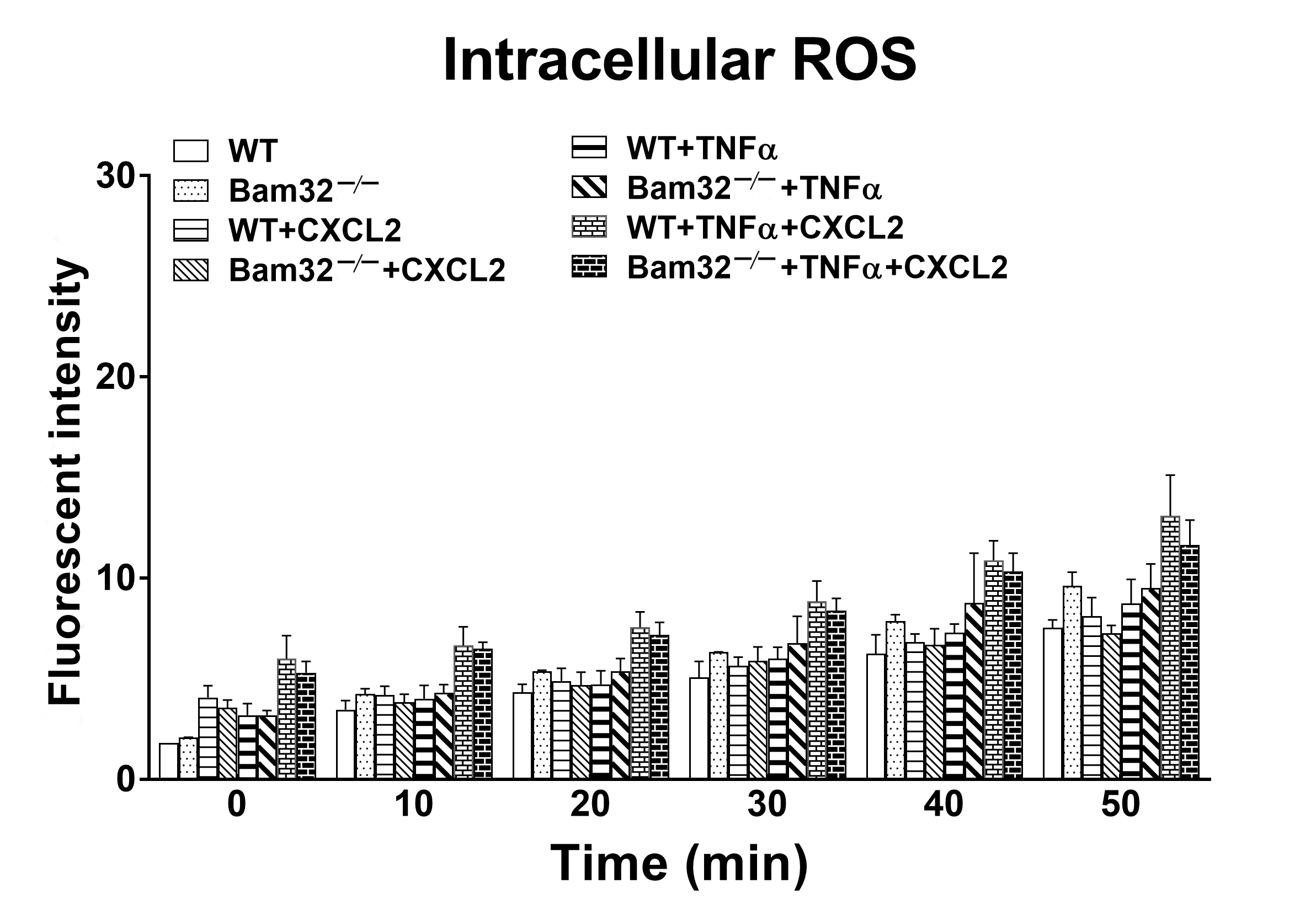

Supplement: Figure S2 — Time course of CXCL2 (0.5 nM)-induced generation of intracellular ROS in isolated neutrophils primed with or without TNFα (12 pM). Mean ± SEM, n = 4. [file Image_2.TIF]

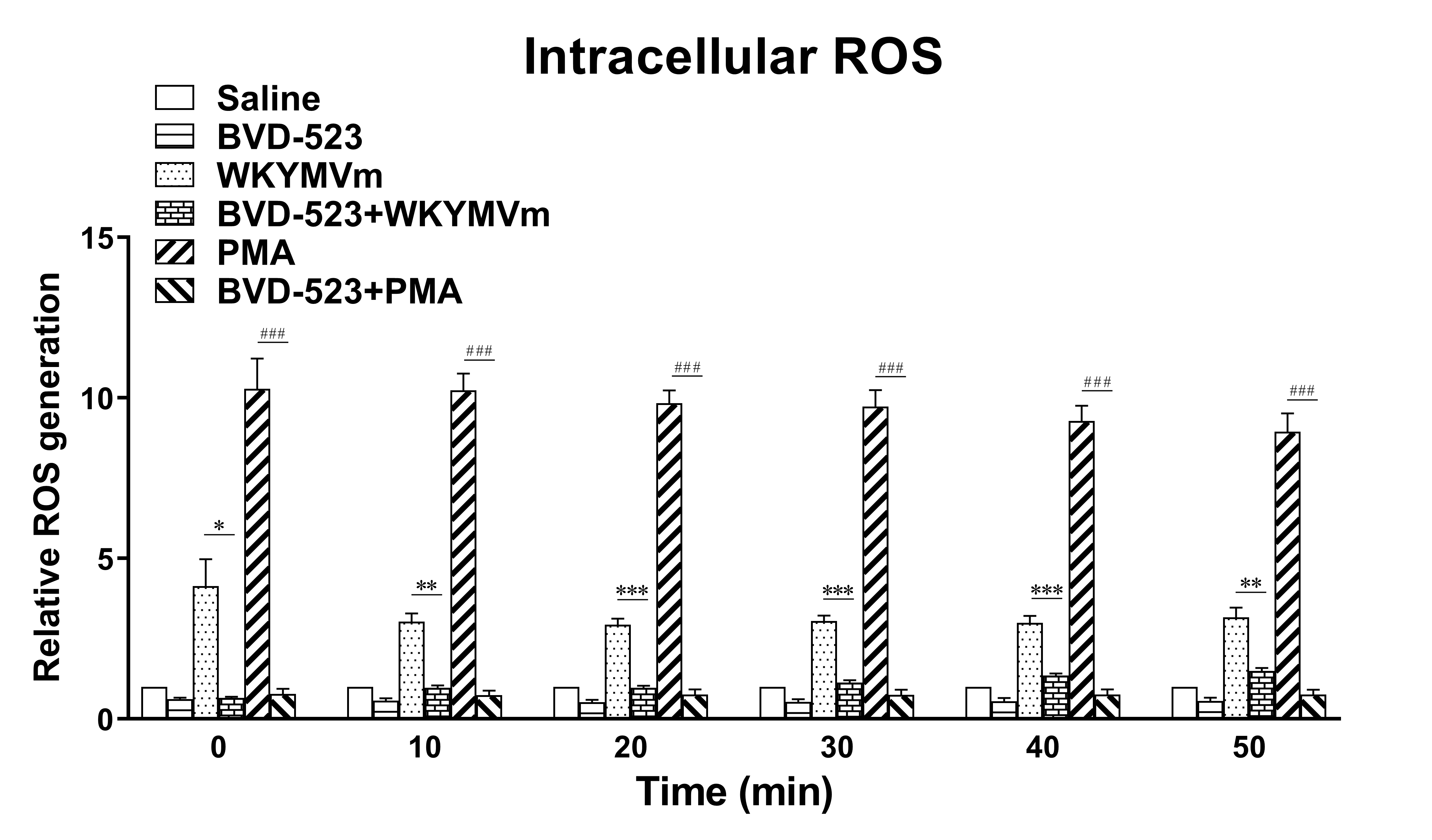

Supplement: Figure S3 — Effects of ERK1/2 inhibitor BVD-523 on intracellular ROS production in WT neutrophils treated with WKYMVm (0.1 μM) or PMA (0.2 μM). Mean ± SEM, n = 3–4. Significant differences between WKYMVm-stimulated groups with and without BVD-523 (*p < 0.05, **p < 0.01 and ***p < 0.001). Significant differences between PMA-stimulated groups with and without BVD-523 (###p < 0.001). [file Image_3.TIF]
